# Vestibulocochlear manifestations in patients with type I diabetes mellitus

**DOI:** 10.1016/S1808-8694(15)30079-3

**Published:** 2015-10-19

**Authors:** Karlin Fabianne Klagenberg, Bianca Simone Zeigelboim, Ari Leon Jurkiewicz, Jackeline Martins-Bassetto

**Affiliations:** a Master’s degree in communications disorders, Tuiuti University, Parana state. Speech therapist; b Doctorate in the sciences of human communications disorders, UNIFESP. Coordinator of the graduate program (master’s degree and doctorate) in communications disorders, Tuiuti University, Parana state; c Doctor in anatomy, UNIFESP. Associate professor in the graduation program (master’s degree and doctorate) in communications disorders, Tuiuti University, Parana state; d Master’s degree in communications disorders, Tuiuti University, Parana state. Speech therapist. Otoneurology unit of the Tuiuti University of Parana state (UTP)

**Keywords:** diabetes mellitus, vestibular diseases, vestibular function test

## Abstract

Glucose metabolism has a significant impact on inner ear physiology, and small changes may result in hearing and balance disorders. **Aim:** To investigate vestibulocochlear symptoms in patients with type I diabetes mellitus. **Study design:** a cross-sectional study of a contemporary group. **Material and Method:** 30 patients referred from Clinical Hospital-UFPR to the Laboratory of Otoneurology-UTP between Mar/2004 to Feb/2005 were evaluated. The following procedures were carried out: a medical history, otological inspections, audiometry, acoustic impedance tests, and vestibular function tests. **Results:** The prevalence of otoneurologic complaints was: headache (23.3%), vertigo (16.6%), and tinnitus (13.3%). The prevalence of associated complaints and habits was: caffeine abuse (20.0%), allergies (10.0%), and alcohol abuse (10.0%). The prevalence of normal auditory thresholds was 90.0%. Acoustic impedance showed no changes. The vestibular test showed changes in 60.0% of cases. Peripheral vestibular deficiency syndromes were also found. **Conclusions:** Significant vestibular system changes were found (60.0%) compared to the auditory system (10.0%). Audiometry revealed mostly normal results. The vestibular test showed changes in the peripheral vestibular system and the peripheral vestibular deficiency syndrome.

## INTRODUCTION

The most important non-transmissible diseases (NTD) for public health in Latin America and the Caribbean region are cardiovascular diseases, cancer and diabetes mellitus (DM). Of these, DM is considered an economic, social and personal burden for institutions and families.^1^ The current world diabetic population is about 135 million people,^2^ a number that might reach 221 million by 2010.^3^ This increase will be significant in Latin America, as 80% of life-years lost due to DM-caused incapacity occur in developing countries.^4^

Estimates for 2025 show that there may be about 11 million diabetics in Brazil, an increase of more than 100% compared to the current number (five million diabetics).^3^

A multicentric study on the prevalence of DM in nine Brazilian capital cities between 1986 and 1988 in the urban population aged between 30 and 69 years revealed that the prevalence of DM is 7.6%. It is believed that a longer life span has led to an increased prevalence of DM.^5^

DM may be defined as a metabolic disorder in which a relative or absolute insulin deficiency causes chronic hyperglycemia.^6^

Metabolic alterations in DM alter the carbohydrate, lipid and protein metabolism in the human body. The disease interferes with the metabolism of glucose and other energy-producing substances.^7^

The etiological classification of glucose disorders by the World Health Organization is based on work done by the National Diabetes Data Group (NDDP) in the United States of America. The NDDP suggests classifying DM and other states of glucose intolerance into three subclasses, as follows: type 1, type 2 and secondary diabetes associated with another identifiable condition or syndrome.^8^

DM is considered the main cause of blindness, of end-stage renal failure and of non-traumatic amputation during the productive age. DM increases the risk of cardiovascular and cerebral diseases 2 to 7-fold, and is also an important cause of neonatal morbidity. Recent data have shown that most of the debilitating complications of the disease may be avoided or delayed by the prospective treatment of hyperglycemia and of cardiovascular risk factors.^8^

Glucose metabolism significantly influences the physiology of the inner ear, which is very active metabolically.^9^, ^10^, ^11^, ^12^, ^13^

The inner ear does not store energy, so minor variations in blood glucose affect its function and cause balance disorders.^9^, ^10^, ^11^, ^12^, ^13^ Altered inner ear metabolism may lead to potassium transfer from the endolymph to the perilymph and an opposite movement of sodium. This mechanism may cause vertigo, tinnitus, hypoacusis and ear fullness.^13^

Various studies^14^, ^15^, ^16^ have shown that both the peripheral and central vestibular systems may be altered in type 1 DM patients.

The aim of this study was to investigate vestibulocochlear manifestations in patients with type 1 DM.

## MATERIAL AND METHODS

Thirty patients (17 males and 13 females) aged between 7 and 56 years (mean age - 25.7 years) with a diagnosis of type 1 DM were assessed.

The study was a cross-sectional contemporary cohort trial in which patients were assessed independently of the type and duration of treatment.

The study was approved by the institutional Research Ethics Committee (protocol number 009/2005).

Patients signed the free informed consent form before undergoing the following procedures:

### Clinical history

A questionnaire was applied, emphasizing otoneurological signs and symptoms, and the personal and family history. Patients with other diseases besides type 1 DM were excluded.

### Otorhinolaryngological evaluation

This assessment was one to exclude conditions that might interfere with auditory tests.

### Audiological evaluation

Conventional pure tone audiometry was done using an Interacoustics AC 40 audiometer and TDH 39P earphones (thresholds in dB NA). The speech recognition threshold was done followed by the percentage rate of speech recognition, in an acoustic booth to avoid interference from extraneous noise. The degree and type of hearing loss were classified according to Davis and Silverman^17^ and Silman and Silverman.^18^

### Acoustic immitance testing

This procedure was done to assess the integrity of the ossicular and the tympanic systems; it is based on the tympanometric curve and investigation of the acoustic reflection. The equipment was an Interacoustics AZ-26 impedance meter and TDH 39P earphones. Jerger’s^19^ criteria were used to interpret the results.

### Vestibular assessment

Patients underwent the following tests that are part of the vestibular assessment based on vectoelectronystagmography (VENG):

### Unrecorded

Open eye unrecorded positional nystagmus was investigated to investigate the presence of nystagmus and/or vertigo associated with bodily changes, based on Brandt and Daroff’s^20^ maneuver. Open eye spontaneous and semispontaneous nystagmus in frontal gazing and at 30° to the right, to the left, upwards and downwards was done.

### Recorded

A Berger VN316 three-channel thermosensitive device was used for measuring VENG. Skin around the orbits was cleaned using an alcohol solution; on each patient an active electrode was placed on the lateral angle of each eye and on the frontal midline, forming an isosceles triangle (electrolytic paste was used for fixation). This setup made it possible to check horizontal, vertical and oblique eye movements. This form of VENG provided us with precise measurements of the slow component angular velocity (vestibular correction) of nystagmus. A Ferrante rotating descending pendular rotating chair, a Neurograff model EV VEC visual stimulator and a Neurograff model NGR 05 air otocalorimeter were used at air temperatures of 42°C, 20°C and 10°C for caloric tests.

The following ocular and VENG labyrinth tests were done according to Mangabeira-Albernaz, Ganança and Pontes’s^21^ criteria:

* calibration of ocular movements: regularity of tracings were assessed to enable comparisons between studies.
* testing of spontaneous nystagmus (open and closed eyes) and semispontaneous nystagmus (open eyes): the presence, direction, inhibiting effect of ocular fixation (IEOF) and the maximum slow component angular velocity (SCAV) of nystagmus were assessed.
* pendular tracking test: the presence and type of curve were assessed;
* optokinetic nystagmus test: the presence, direction, maximum SCAV with clockwise and anticlockwise movement of the light source were assessed, and the preponderant direction of nystagmus was calculated.
* investigation of pre- and post-rotatory nystagmus by the pendular swing rotatory test with stimulation of the anterior, lateral and posterior semicircular canals: the presence, direction, frequency after anticlockwise and clockwise rotation and calculation of the preponderant direction were noted.
* investigation of pre- and post-caloric nystagmus: done with the patient’s head and trunk tilted backwards by 60° for adequate stimulation of the lateral semicircular canals. Stimulation time for each ear was 80 sec per ear at each temperature (42°C, 20°C and 10°C) and responses were recorded with eyes closed and then with eyes open to observe IEOF. The direction, absolute values of SCAV and calculation of the preponderant direction and labyrinthic predominance of post-caloric nystagmus.

## RESULTS

Patient complaints, the clinical history and habits are shown on Tables 1 and 2.

**Table 1:** Frequency of otoneurological complaints in the clinical history of 30 patients with type 1 diabetes mellitus.

Otoneurologic Complaints		
	N. Of Patients	Frequency (%)
Headache	7	23.3
Rotating Dizziness	5	16.6
Tinnitus	4	13.3
Listening Difficulties	3	10.0
Non-rotating Dizziness	2	6.6
Ear Pain	1	3.3
Sensitivity To Sound	1	3.3

**Key:** N. - number.

**Table 2:** Frequency of associated complaints, personal history and habits in the clinical history of 30 patients with type 1 diabetes mellitus.

Associated Complaints		
	N. Of Patients	Frequency (%)
Caffeine Abuse	6	20.0
Allergy	3	10.0
Alcohol Abuse	3	10.0
Low Blood Pressure	2	6.6
Smoking	2	6.6
Tingling Of Extremities	2	6.6
Nightmares	1	3.3
Darkened Vision	1	3.3
Sweating	1	3.3

**Key:** N. - number.

Audiological tests revealed alterations in three cases (10 %), as follows: one case of flat bilateral moderate sensorineural hearing loss and two cases of descending bilateral moderate sensorineural hearing loss, as shown on Table 3.

**Table 3:** Audiological findings in 30 patients with type 1 diabetes mellitus.

Findings	N. Of Patients	Frequency (%)
N.a.t.	27	90.0
Descending Bilateral M.s.h.l.	2	6.7
Flat Bilateral M.s.h.l.	1	3.3

Total	30	100.0

**Key:** N.A.T. - normal auditory thresholds; descending bilateral M.S.H.L. - descending bilateral moderate sensorineural hearing loss; flat bilateral M.S.H.L. - flat bilateral moderate sensorineural hearing loss; N. - number.

Tests for vertigo and/or positional nystagmus, calibration of ocular movements, spontaneous nystagmus (open and closed eyes), semispontaneous nystagmus (open eyes), pendular tracking, optokinetic nystagmus and per-rotatory nystagmus were within normal limits. Altered VENG results were those of the caloric test.

Vestibular function testing revealed changes in the peripheral vestibular system in 18 cases (60 %). The vestibular exam was within normal limits in 12 cases (40 %).

Absolute and relative values of caloric stimulation testing are shown on Table 4.

**Table 4:** Caloric stimulation testing - post-caloric nystagmus (absolute and relative values) in 30 patients with type 1 diabetes mellitus.

Findings	N. Of Patients	Frequency (%)
Normoreflexia	12	40.0
Bilateral Labyrinthic Hyporeflexia	10	33.3
Unilateral Labyrinthic Hyporeflexia	2	6.7
Bilateral Labyrinthic Hypereflexia	5	16.7
Unilateral Labyrinthic Hypereflexia	1	3.3

Total	30	100.0

**Key:** N. - number.

Results of vestibular testing were as follows: 12 cases (40 %) were within normal limits, 12 cases (40 %) presented the peripheral vestibular syndrome, and 6 cases (20 %) presented irritative peripheral vestibular syndrome, as shown on Table 5.

**Table 5:** Results of vestibular testing in 30 patients with type 1 diabetes mellitus.

Findings	N. Of Patients	Frequency (%)
N.v.t.	12	40.0
D.p.v.s.	12	40.0
I.p.v.s.	6	20.0

Total	30	100.0

**Key:** N.V.T. - normal vestibular test; D.P.V.S - deficient peripheral vestibular syndrome; I.P.V.S. - irritating peripheral vestibular syndrome; N. - number.

Vestibular alterations were found in 18 cases (60 %) and auditory alterations were found in 3 cases (10 %). Of these, two cases had both of these changes.

## DISCUSSION

Patients with altered glucose metabolism may present auditory and vestibular signs and symptoms. The most prevalent otoneurological complaints in our study were as follows: headache - seven cases (23.3%), rotatory dizziness - five cases (16.6%) and tinnitus - four cases (13.3%). Various published papers^22^, ^23^ have reported these symptoms in metabolic disease. The inner ear is particularly sensitive to altered blood glucose and insulin levels; the most common symptoms are vertigo, hearing loss, tinnitus and ear fullness, among others. Vascular striae depend on a constant concentration of blood glucose; variations of blood glucose may cause auditory and balance disorders.^13^

The most prevalent associated complaints and other referred habits were the following: caffeine abuse - six cases (20 %), allergy - three cases (10 %) and alcohol abuse - three cases (10 %). Ganança, Dias and Ganança^24^ noted that caffeine acts as a diuretic and its stimulating effect may worsen symptoms such as vertigo and tinnitus. Alcohol abuse may directly affect the inner ear by changing the concentration of its liquids; this effect may cause or augment cochleovestibular symptoms.

Vestibular alterations were seen in 18 cases (60 %) and hearing loss was observed in three cases (10 %). Of these, two cases had both disturbances. Metabolic disorders may affect the homeostasis of the vestibular organ more rapidly than the auditory system.^16^ Gawron et al.^16^ noted that vestibular testing appeared to be more sensitive in detecting central nervous system disorders in DM patients compared to audiological testing. In this same paper, the authors found that auditory tests and acoustic immitance testing were within normal limits in these patients, even though most of them revealed central alterations when assessed by vestibular testing.

Maia and Campos^25^ stated that there is no consensus between audiological and histopathological findings in type 1 DM. We found three cases (10 %) of sensorineural hearing loss; one of theses cases (3.3%) had flat bilateral moderate sensorineural hearing loss, and two cases (6.6%) had descending bilateral moderate sensorineural hearing loss. Clinical trials have revealed a relation between hearing loss and DM. Camisasca^26^ found sensorineural hearing loss in 46% of cases, although the degree and configuration were not mentioned in this study. Jorgensen and Buch^27^ reported bilateral sensorineural hearing loss in 41% of cases. Khasanov, Vasilyeva and Mazovetsky^14^ reported hearing loss and vestibular involvement in 18 cases (43 %). Biurrun et al.^15^ found 11 cases (23.9%) of mild sensorineural hearing loss mostly at high frequencies, with no associated auditory complaints. Almeida^28^ found five cases (20 %) with sensorineural hearing loss (unreported degree) in a study of patients with glucose metabolic disorder.

Inner ear blood vessel involvement and alterations of the vascular striae in DM patients have been established by various authors to suggest a connection between hearing loss and DM. These changes strongly suggest that DM causes hearing loss.^27^, ^29^, ^30^, ^31^, ^32^

We found 18 cases (60 %) with altered vestibular tests. These findings are similar to published results^15^, ^6^, ^33^, ^34^ that have also shown altered vestibular tests.

Biurrun et al.^15^ have suggested that recently diagnosed DM patients show no vestibular test abnormalities. They raised the hypothesis that the effect of DM on vestibular function might be caused by complications such as diabetic neuropathy and angiopathy, which are absent in initial phases of the disease. Various authors^27^, ^29^, ^30^, ^31^ have previously suggested that microangiopathy might be responsible for DM-associated altered inner ear function.

Vestibular testing is essential for investigating bodily balance; the importance of caloric stimulation testing is its ability to assess each labyrinth separately.

Abnormal VENG findings were uncovered in caloric stimulation testing. Other tests, such as the presence of vertigo and/or positional nystagmus, calibration of ocular movements, open and closed eye spontaneous nystagmus, open eye semispontaneous nystagmus, pendular tracking, optokinetic nystagmus and per-rotating nystagmus revealed no changes. Scherer and Lobo34 found altered electronystagmographies in 87.5% of subjects; in 12.5% of cases the diagnosis was based on positional nystagmus. Biurrun et al.^15^ found seven cases (15.2%) with spontaneous nystagmus and 12 cases (26.1%) with positional nystagmus. Almeida28 found over 50% of altered caloric stimulation tests; pendular tracking was the second test with most alterations.

We found 12 cases (40 %) of normoreflexia, 12 cases (40 %) of hyporeflexia and 6 cases (20 %) of hypereflexia. These findings are similar to those of Biurrun et al.,^15^ Camisasca,26 and Aantaa and Lehtonen,^33^ who noted that hyporeflexia is more prevalent that hypereflexia, different from studies by Scherer and Lobo,^34^ and Cojazzi.^35^ Mangabeira-Albernaz^12^ contend that both hypereflexia and hyporeflexia may occur, which would explain the paucity of crises of vertigo.

We found 18 cases (60 %) of peripheral vestibular involvement, of which 13 cases (43.3%) had no dizziness, similar to findings by Ramos et al.10 who stated that DM patients using insulin have less dizziness, which would explain the low rate of dizziness in our sample. Jerger and Jerger^36^ contend that 20% of DM patients may present dizziness. Some patients, however, have no complaints. Scherer and Lobo^34^ found altered vestibular function in 75% of subjects, with 62.5% presenting no otoneurological complaints. According to Munhoz et al.,^22^ absent vestibular symptoms associated in the presence of altered vestibular tests may be a sequelae of a previous disorder that is not always evident in the clinical history; active vestibular disease may also be a possible explanation.

Peripheral vestibular involvement was found in 100% of the 18 cases that had altered vestibular function. This finding diverge from those in studies by Khasanov, Vasilyeva and Mazovetsky,14 and Gawron, Pospiech, Orendorz-Fraczkowska and Noczynska16 who observed that metabolic disorders found in type 1 DM caused changes in different segments of the vestibular organ, but mostly in the central portion.

## CONCLUSION

- Otoneurological complaints included headache (23.3%), rotating dizziness (16.6%) and tinnitus (13.3%). Associated complaints and habits included caffeine abuse (20 %), alcohol abuse (10 %) and allergy (10 %).
- There were more changes in the vestibular system (60 %) compared to the auditory system (10 %). Auditory testing was mostly within normal limits. Vestibular testing revealed alterations in the peripheral vestibular system and the deficient peripheral vestibular syndrome.
